# Endoparasites of Selected Native Non-Domesticated Mammals in the Neotropics (New World Tropics)

**DOI:** 10.3390/vetsci6040087

**Published:** 2019-10-30

**Authors:** Kegan Romelle Jones, Kavita Ranjeeta Lall, Gary Wayne Garcia

**Affiliations:** 1 The Department of Basic Veterinary Sciences (DBVS), School of Veterinary Medicine (SVM), University of the West Indies (UWI), Mt. Hope, Trinidad and Tobago; 2 The Open Tropical Forage-Animal Production Laboratory (OTF-APL), Department of Food Production (DFP), Faculty of Food and Agriculture (FFA), The University of the West Indies (UWI), St. Augustine, Trinidad and Tobago; k_lee_24@yahoo.com (K.R.L.); prof.gary.garcia@gmail.com (G.W.G.)

**Keywords:** *Dasyprocta leporina*, *Agouti paca*, *Hydrochoerus hydrochaeris*, *Didelphis marsupialis insularis*, *Tayassu tajacu*, *Peccari tajacu*, *Mazama amaericana*

## Abstract

In this review, information was summarized on endoparasites found in six non-domesticated neotropical animals. These mammals have the potential to be domesticated. The animals included three rodents, agouti (*Dasyprocta leporina*), lappe (*Agouti paca*), and capybara (*Hydrochoerus hydrochaeris*); a marsupial, manicou (*Didelphis marsupialis insularis*); and an artiodactyl, the collared peccary (*Tayassu tajacu*/*Peccari tajacu*) and a ruminant (the red brocket deer, *Mazama americana*). While there are many descriptions of the parasites present, the majority of publications failed to note the effect of them on the animals. Most information is available on endoparasites of capybara, while the endoparasites of the red brocket deer were the least reported. The manicou was reported to have had the most number of endoparasites, 44 species of parasites were reported, while there were only 24 endoparasites reported in the lappe. The most common parasites found in these neotropical animals were *Paraspidodera uncinata*, *Strongyloides* spp., *Eimeria* spp., *Moniezia benedeni*, *Trichuris* spp., *Physocephalus* spp., and *Giardia* spp. A large majority of the studies concluded that these animals were reservoirs for parasites that could affect domesticated livestock. Endoparasites of zoonotic significance were *Echinoccocus* spp., *Trichuris* spp., *Giardia* spp., and *Cryptosporidium* spp.

## 1. Introduction

In this review, updates were made on the current knowledge of endoparasites in non-domesticated animals with the potential for domestication. These animals have the potential to be meat protein sources for rural communities. Former reviews on endoparasites in the neotropics summarized the information on cattle (*Bos indicus* and *Bos taurus*), sheep (*Ovis averies*), goats (*Capri hircus*), horses, (*Equus caballi*), pigs (*Sus sucrofa*), and chickens (*Gallus domesticus*) [1]. Endoparasites mainly found in these domesticated animals were *Strongylus vulgaris* in horses, *Hyostrongylus rubidis* in pigs, *Haemonchus contortus* in ruminants, and *Heterakis gallinarum* in chickens. Some endoparasites of ducks (*Anas platyrhynchos* and *Cairina moschata*), turkeys (*Melaegradis gallopavo*), chinchillas (*Chinchillas lanigera*), guinea pigs (*Cavia porcelleus*), and south american camelids (*Lama glama*, *Lama pacos*, *Lama gunacoa*, and *Vicuna vicuna*) were *Nematodirus* spp., *Trichostrongylus* spp., *Giardia duodenalis, Cryptosporidium* spp., and *Ascaridia gallinarum* [2].

Since these six native neotropical animals have potential for domestication [3], it is required to focus on factors which affect animal production. These factors are (i) feeding and nutrition, (ii) reproduction and breeding, (iii) health and disease, (iv) housing and environement, (v) economic and socioeconomic factors, and (vi) animal behavior and psycology [3,4]. Recently, the agouti, *D. leporina*, has been studied in more detail, with focus on the anatomy of the male and female reproductive system, oestrus cycle in the female (28–31 days) as well as semen analysis using electroejaculation [5,6,7,8] Further anatomical investigations of the digestive system of the agoutis revealed that the majority of the gastrointestinal tract was small intestine in conjunction with a large cecum [9]. The agoutis mainly are frugivorous with the majority of the diet consisting of nuts and fruits, with potentially a preferential particle size [10,11,12,13]. Earlier it was found that these animals were rather omnivorous and would consume eggs and chicks [14]. It is clear that there is a need for more investigation on their feeding habits. Several studies have reported on the blood values of captive reared animals, and differences in diets and environments did not affect those values [15]. As such, blood values are a good basis for determination of the health status of neotropical mammals.

The lappe was decribed as a frugivore, the manicou as an omnivore, the collared peccary as a pseudo-ruminant herbivore, and the red brocket deer was grouped with ruminants [12,13,14,15,16,17,18,19]. The manicou has a gestation period of three weeks [17,18]. Other information on these species is lacking. The objective of this review was to summarize the knowledge of endoparasites found in six native non-domestic neotropical animal species. These neotropical animals are potential sources of meat protein which can be utilized by rural communities.

## 2. Agouti (*Dasyprocta* spp.)

The earliest work on the gastrointestinal parasites of the agouti, *D. leporina*, was done in the mid 1930s. *Strongyloides agoutii* was described in the feces of the agouti. This parasite has a similar life cycle as the other species of the *Strongyloides* genus [20]. Similar work was done on agoutis found in Trinidad, but the entrails of the animals were analyzed for gastrointestinal parasites. In the early 1950s, the amphistome, *Stichorchis giganteus*; the tapeworm, *Raillietina demerariensis* var. *trinitatae*; and seven roundworms including *Strongyloides agouti*, *Trichuris gracilis* var. *trinitatae*, *Acanthocheilonema* spp., *Fuellebornema agoutii*, *Pudica pudica*, *Aspidodera binansata* var. *agoutiae*, and *Helminthoxys urichi* were detected in the agouti [21].

In presumed healthy wild agoutis from Trinidad, *Trichuris gracilis* var. *trintatae* (in cecum and large intestines), *Strongyloides agouti*, *Helminthoxys urichi*, *Eimeria* spp., ascarid-like ova (unidentified species), trematode (unidentified species), and an unidentified cestode were detected [22] and more recently in intensively reared agouti (*D. leporina*), *Strongyloides* spp. was found in 36.9%, *Trichuris* spp. in 4.62%, and *Paraspidodera uncinata* in 15.4% of the agoutis sampled (n = 65). Despite the presence of these parasites, the animals were in good body condition (average body condition score of 3 out of 5) and had no history of gastrointestinal illness [23]. Using fecal floatation techniques, *Trichuris* spp. were found at a concentration of 2.2 × 10^2^ eggs/gram, *Strongyloides* spp. at 4.28 × 10^2^ eggs/gram, and *Eimeria* spp. at 2.15 × 10^3^ oocysts/gram, all with no signs of gastrointestinal illness and a body condition score of 3 out of 5 [24].

In Paraguay, wild agoutis (*D. azarae*) were positive for *Pudica pudica*, *Pudica gonosoma*, *Durettestrongylus baudi*, *Fuellebornema granulosa*, and *Fuellebornema demarsae* [25]. In several studies in Brazil, agouti parasites that were found included *Trichuris* spp. (cecum), *Eucyathostomum copulatum*, *Helminthoxys urichi* (cecum and intestines), *Physocephalus meridionalis* (large intestine) and *Dipetalonema* spp. (body cavity), *Eucyathostomum copulatum* (large intestine), *Heligmostrongylus agouti*, *H. almeidai*, *Heligmostrongylus sedecimradiatus*, *Monodontus aguiari*, *Pudica pudica*, *Pereiraia* spp., *Physocephalus meridionalis*, *Trichuris gracilis* (cecum), *Raillietina trinitae* (small intestine), *Physaloptera toressi*, *P. mediospiralis* (stomach), *Freitastrongylus angelae* (stomach), *Vianella trichospicula*, and *Avellaria intermedia* were found [26,27,28,29,30,31,32,33,34,35]. The *Eimeria* spp. identified were *E. aguti*, *E. cotiae*, and *E. paraensis*. The predilection site of these protozoan parasites was the small intestines, but none of the infected animals showed signs of illness and the fecal pellets found were of normal consistency [31]. Reported prevalences were 45.3% (24/53) for protozoan oocysts, 10.8% (7/53) for *Trichuris* spp. eggs, and 18.8% (10/53) for *Strongyloides* spp. [29].

In a zoo in Mephis Tennessee, *Echinococcus oligarthrus* hydatid cysts and *Strongyloides* spp. were found in healthy Brazilian agoutis from Guyana. These animals were treated with ivermectin (0.7 mg subcutaneously, twice daily for 14 days), praziquantel (28.4 mg intramuscularly, twice daily for 30 days), and albendazole (65 mg per os, once a day for 30 days) and some of the subcutaneous cysts were removed surgically [36]. In the studies performed in Argentina, similar parasites as to those mentioned above were found [37].

Other treatments that were reported included fenbendazole (5 mg/kg/day for 5 days) and piperazine citrate (180 mg/kg/day for 5 days), and the efficacy reported with these treatments were poor [38]. Most authors identified various parasites in captive-reared and wild agoutis in the neotropical region; however, only a few made reference to the effect these parasitic organisms had on the animals’ health and body condition. A summary of the endoparasites found in the agouti at different locations and the effects on the animals (host) can be seen in Table 1.

## 3. Lappe (*Agouti paca*/*Cuniculus paca*)

Most of the studies on lappe were from Brazil and the following parasites were found: Heligmostrongylus sedecimradiatus, *Longistriata breviscapula*, *Oswaldonema cruzi*, *Oswaldonema skrjabini*, *Paraspidodera uncinata*, *Paraspidodera* spp., *Pereiraia* spp., *Turgida torresi*, *Trichuris* spp., and *Vianella avellari*. *Physocephalus* spp. was found in the stomach, and *Strongyloides* spp. and *Strongylus* spp. were found in the small intestine. *Heligmostrongylus agouti* and *Physocephalus meridionalis* were found in the stomach and *Echinococcus vogeli* and *Calodium hepaticum* were found in the liver [26,27,39].

Few studies reported the pathology casued on internal organs by the endoparasites. The liver pathology potentially caused by *Echinococcus vogeli* and *Calodium hepaticum* was characterized by fibrosis with a scanty infiltrate of lymphocytes and macrophages [39] and the histopathological lesions of *Physocephalus* spp. in the stomach showed congestion, interstitial hemorrhaging, and mononuclear inflammatory infiltrates [40]. In a breeding farm in Brazil, *Eimeria* spp., *Strongyloides* spp., *Trichuris* spp., and *Hymenolepis diminuta* were found and managed by anthelmintics and sanitation [41]. Quick lime was also used as an anthelmintic treatment for the larval stages of the parasites in the soil [42].

In a few other countries, the *Strongyloides* spp. were found [43]. In Costa Rica, *Strongyloides* spp., Strongylida, *Eimeria agouti*, *Capillaria* spp., Ascaroidea, *Taenia* spp., and *Trichuris* spp. were found in the gastrointestinal tracts [44]. In Mexico, *Strongyloides* spp., Strongylida, *Trichuris* spp., and Eucoccidia were found [45] and in Venezuela, *Raillietina demerariensis* (a cestode) was found [46].

*Echinococcus* spp. take a special place in the list of parasites found in the lappe as they are zoonotic. They have been found in Columbia where approximately 47% (44/93) of the lappe were found to be infected [47]. In Bolivia as well as Peru, *Echinococcus vogeli* was found but there were no relyable prevalence figures available [48,49,50]. A later study in Peru demonstrated larval cysts of *E. vogeli* in 12% of the lappe (15/120) [51].

Echinococcosis has been reported as being zoonotic and the agouti (*D. leporina*) and the lappe (*A. paca*) are the intermediate hosts of *E. vogeli* and *E. oligarthrus*. However, humans become infected via the ingestion of eggs that come from the adult parasites present in dogs [51,52,53,54,55,56]. All data on endoparasites in the lappe are summarized in Table 2.

## 4. Capybara (*Hydrochoerus hydrochaeris*)

The first reports that were made in Panama on endoparasites was in the 1930s and amphistomate flukes (*Taxorchis schistocotyle*) were detected in a dead animal. However, no lesions were found in the intestinal tract. [57]. Another case report came from the San Diego Zoo, where *Balantidium coli* was associated with mortality as well as mucoid diarrhea. Histological findings of the colon found ulceration and cellular infiltrates that consisted of eosinophils, macrophages, and plasma cells [58]. In Brazil, the death of a capybara was associated with four specimens of trematodes, Trichostrongylidae, *Eimeria*, and *Strongyloides* spp. There was multifocal fibrosis with marked thickening of the liver capsule and granulomatous thickening of the bile ducts [59]. *Eimeria ichiloensis* and *Eimeria trinidadensis* have been associated with diarrhoea in the capybara [60].

In Brazil, numerous studies have been conducted on the gastrointestinal parasites of capybaras. *Taxorchis schistocotyle*, *Cruorifilaria tuberocauda*, *Eucoleus hydrochoeri*, *Echinocoleus hydrochaeri*, *Habronema clarki*, *Hydrochoerisnema anomalobursata*, *Protozoophaga obesa*, *Strongyloides chapini*, *Trichostrongylus axei*, *Vianella hydrochoeri*, *Yatesia hydrochoerus*, *Monecocestus hagmanni*, *M. hydrochoeri*, *M. macrobursatum*, *M. hagmani*, and *H. fuelleborni* have been found in capybaras. *Capillaria hydrochoeri*, *Philophthalmus lachrymosus*, *Hippocrepis hippocrepis*, *Neocotyle neocotyle*, *Fasciola hepatica*, *Nudacotyle tertius*, *Nudacotyle valdevaginatus*, *Taxorchis schistocotyle*, *Eimeria ichiloensis*, *Fasciola hepatica*, *Eimeria* spp., cestodes, Trichostrongylidae, *Capillaria* spp., Anoplochephalidae, Ascaridae, and *M. jacobi* have also been found [27,35,61,62,63,64,65,66,67,68].

Three more detailed studies on both juvenile and adult capybaras located the helminths at their predilection sites: *Trichostrongylus axei* (stomach and small intestines), *Vianella hydrochoeri* (stomach and small intestines), *Strongyloides chapini* (stomach and small intestines), *Yatesia hydrochoerus*, *Cruorifilaria tubercauda*, and *Capillaria hydrochoeri* (stomach and small intestines), *Protozoophaga obesa* (pyloric region of the stomach, cecum, colon, and rectum), *Taxorchis schistocotyle* (small and large intestines including cecum), *Hippocrepis hippocrepis* (small intestine, colon, and rectum), *Nudacotyle tertius* and *Monoecocestus hydrochoeri* (small intestines), *Hydrochoerisnema anomalobursata* (stomach and small intestine), *Monoecocestus hagmanni* (small intestine), *M. macrobursatum* (small intestine), *Monoecocestus hagmanni* (small intestine), and *Philophthalmus lachrymosus* (vitreous humour). *Stongyloides* spp. (stomach), *Habronema* spp. (stomach), *Vianella hydrochoeri* (stomach and small intestine), *Hydrochoeristrema cabrali* (cecum) [66,69,70,71] and *S. chapini* had a higher prevalence in juvenile animals in comparison with adults, while for *Y. hydrochoerus* and *C. hydrochoerid*, the opposite was recorded [69].

A more epidemiology-based study on 45 samples showed that 5.52% of the samples were positive for *C. parvum* [72], while another study showed that, of the 250 samples tested, 52.4% were positive for *Eimeria trinidadensis*, *Eimeria ichiloensis*, *Eimeria boliviensis*, and *Eimeria araside* [73]. Nine percent of 134 capybaras were found positive for *Fasciola hepatica* [74]. In 31 free ranging capybaras, 58% were positive for *Protozoophaga* spp., 23% for *Vianella* spp., 10% for *Strongyloides* spp., and 10% for Ancylostomatidae [75]. In Argentina, about 70% of the capybaras were found positive for endparasites and infections with multiple parasites and protozoa were higher in young animals and in spring. Parasites found included: *Eimeria* spp., *Blastocystis* spp., *Balantidium coli*, Ascaridia, Trichostrongylidae, *Protozoophaga obesa*, *Strongyloides* spp., *Capillaria hydrochoaeri*, *Monoecocestus hydrochoerid*, and *Taxorchis schistocotyle* [76]. Other studies in Argentina found *Fasciola hepatica, Echinocoleus hydrochoerid*, and *Taxorchis schistocotyle* [77,78]. In 2019, a new species named *Trichuris cutillasae* was identified in the cecum of capybaras [79].

In Bolivia, in the mid 1990, helminths including *Monoecocestus hagmanni*, *M. hydrochoeri*, *M. macrobursatum*, *Habronema clarki*, *Vianella hydochoeri*, *Protozoophaga obesa*, *Taxorchis schistocotyle*, and *Hippocrepis hippocrepis* were found [80], while the protozoa found were *Eimeria trinidadensis*, *E. ichiloensis*, and *E. boliviensis* [81]. A more detailed study in Venezuela found a negative association between the body condition and helminth intensity for *M. macrobursatum* (found in the small intestine), *V. hydrochoeri* (small intestine), and *H. hippocrepis* (large intestine) [82]. Other parasites found were *Monoecocestus hagmanni* (small intestine), *Protozoophaga obesa* (cecum), *Taxorchis schistocotyle* (cecum), *Hippocrepis hippocrepis*, *Eimeria trinidadensis*, *E. ichiloensis*, and *E. boliviensis* [81,82]. In Columbia, the following protozoan parasites were identified: *Sarcocystis* spp., *Eimeria* spp., *Giardia* spp., *Cycloposthium hydrochaeri*, *C. incurvum*, *C. minutum*, *C. lenticularis*, and *C. compressum* [83].

Stress by feed restriction and physical restraint had a negative effect on growth and body condition and induced a significantly higher coccidia infestation, and a lower helminth burden showing that the living conditions may have an influence on parasitic infestation [84]. Environmental conditions such as season, age, and sex of the animals have also been associated with different parasitic infestations. This shows that the interactions amongst gastrointestinal parasites are complex and further research is needed [85].

Capybaras have been shown to have a wide range of endoparasites, but they appear largely resistant to their effects and show only a few signs of ill health [86]. The capybaras, being the largest rodents on earth, have been given a lot of attention. However, the majority of investigations only noted the presence or absence of endoparasites without making observations on the effects these organisms had on the animals (Table 3).

## 5. Manicou (*Didelphis marsupialis insularis*/*Didelphis marsupialis*)

The earliest data on endoparasites of the manicou (*D. marsupialis insularis*) were recorded in the early 1950s in Trinidad. In the large intestine, *Aspidodera raillieti*, *Sublura trinitatis*, *Trichuris reesali*, *Trichuris minuta Trichuris urichi*, *Cruzia cameroni*, *Longistriata didelphis*, and *Viannnaia hamata* were identified. In the small intestine, *Fuellebornema agouti*, *Delicata* spp., *Camerostrongylus didelphis*, and *Capillaria* spp. were recorded. *Physaloptera turgida* and *Spirocerca cylicola* (later found to be *Didelphonema longispiculata* [87]) were found in the stomach and small intestine. While, *Helminthoxys urichi* was found in the stomach [88]. The trematodes *Achillurabainia recondita* and *Rhopalias coronatus* were found in the intestines and *Metadelphis evandroi* was found in the bile duct [89].

In the US (*Alabama* and Georgia), *Isospora boughtoni* and *Isospora* spp. oocysts, *Brachylaima virginianum*, and *Cruzia americana* were found in the intestines of opossum (*D. marsupialis*) [90]. *Phyaloptera turgida* was found in the stomach and ulcerations penetrated from the glandular mucosa to the submucosal level [91]. In the lung, *Capillaria aerophila* and metastrongyloid nematodes which resembled *Perostrongylus* and *Didelphostrongylus hayesi* were found [91,92]. The lungs infested with *Perostrongylus* showed a diffuse interstitial pneumonia pattern [91].

In Brazil, two hundred and twenty-four *D. marsupialis* were examined and fifteen animals were found with *Besnoitia* spp. in cystic leasions in the muscles and viscera [93]. Other parasites detected in Brazil were *Capillaria* spp. (palate), *Eucoleus fluminensis* (mouth), *Trichuris minuta* (large intestine), *Heterostrongylus* spp. (lungs), *Mammomonogamus laryngeus* (nostrils), and *Aspidodera raillieti* (large intestine). *Aspidodera* spp., *Cruzia tentaculata*, *Turgida turgida* (stomach), *Thelazia iheringi*, *Dipetalonema* spp. (liver cysts, peritoneum, skin), *Litomosoides* spp. (skin), *Lagochilascaris turgida, Viannaia hamata*, and *Sarcocystis neurona* [26,27]. *S. neurona* caused neurological diseases in different animal species and is also zoonotic [94]. Similar endoparasites that were found in *D. marsupialis* in Brazil were also found in Peru [95].

In Costa Rica, the coccidian, *Eimeria marmosopos*, was found [96], and this coccidial species was located in the epithelial cell of the small intestines and caused cellular necrosis [97]. Recently in Mexico, the gall bladder was found to be the site of infection for *Amphimerus caudaletestis* and *Philandrophilus magnacirrus*. In the intestines, *Rhopalias coronatus*, *R. macracanthus*, *Thaumasioscolex didelphidis*, *Viannaia viannai*, *Travassostrongylus* spp., and *Onicola luehei* were found and the cecum was the predilection site for *Cruzia tentaculata* and *Trichuris didelphis*. *Turgida turgida* was found in the stomach and *Capillaria* spp. was found in the lungs [98]. *Aspidodera* spp. and *C. tentaculata* were found in the small intestines of *D. marsupialis* in Pocone, Brazil [35] (Table 4).

## 6. Collared Peccary (*Pecari tajucu*/*Tayassu tajacu*)

The earliest study on parasites in peccaries was done in the 1930s in Texas and *Moneizia bendeni* and *Parostertagia heterospiculum* was found in the small intestine [99]. In the stomach, *Physocephalus sexalatus* was found and in the oesophagus, *Parabronema* spp. [99,100]. Much later and in the same region, *Eimeria chaparralensis*, *Eimeria dicotylensis*, *Eimeria pecari*, *Eimeria* spp., *Klossia* spp., *Balantidium* spp., *Dirofilaria acutiuscula*, *Gongylonema baylisi*, *Gongylonema pulchrum, Physocephalus sexalatus, Parabronema pecariae* (Stomach), *Parostertagia heterospiculum*, *Physocephalus* spp., *Texicospirura turki*, *Moniezia benedeni*, and *Fascioloides magna* were found [101,102,103,104,105], while another study failed to recover endoparasites [106]

In Brazil, in the late 1930s, *Gonglyonema baylisi* was found in the oesophagus [107] and later on *Texicospiruria turki*, *Parabronema pecariae*, *Physocephalus sexalatus*, *Gongylonema* spp., and *Gongylonema baylisi* detected in the stomach. *Monodontus semicircularis* and *Moniezia benedeni* in the small intestine; *Eucyathostomum dentatum*, *Trichuris* spp., and *Balantidium coli* in the large intestine; and *Dirofilaria acutiuscula*, *Molineus semicircularis*, *Nematodirus molina*, and *Oesophagostomumdentatum* [27,108,109]. In Peru, eggs of *Ascaris* spp., ancylostomatids, spirurids, *Paragonimus* spp., *Giardia* spp., *Cryptosporidium* spp., *Balantidium* spp., and *Eimeria* spp. were identified in the fecal samples [110,111]. It should be noted that *Cryptosporidium* spp. and *Giardia* spp. are of zoonotic importance (Table 5).

In Surinam, *Toxocara alienata* were found but was not associated with poor body condition [112]. In Mexico, *Globocephalus urosubulatus*, *Parabronema pecariae*, *Parostertagia* spp., *Texicospiruria turki*, and *Paramphistomum* spp. were detected in the stomach and *Oesophagostomum* spp. *Strongyloides* spp., *Eimeria* spp., *Isospora* spp., *Oesophagostomum* spp., and *Moniezia benedeni* inhabited the intestines [113,114]. In Panama, *Entamoeba coli*, *Entamoeba* spp., *Cryptosporidium* spp., *Endolimax nana*, and *Strongyloides* spp. were detected [115]. In Bolivia, *Texicospiruria turki*, *Monodontus angulari*, *Eucyathostomum* spp., *Stichorchis giganteus*, *Moniezia benedeni*, *Ascaris* spp., and *Eimeria* spp. were detected [116]. In the work done in mid 1980s by Hellgren et al. [117] on collared peccaries with signs of respiratory distress and coughing, *Ascaris suum* was found in the bile duct and the same parasite was found in South America [117,118,119].

## 7. Red Brocket Deer (*Mazama americana*)

In Trinidad, in the 1930s, *Mazama simplicicornis* was found to have the following parasites; *Mazamanema longibursatum*, *Ierestrongylus filiformis*, *Mazamastrongylus trinitatis*, *Paramphistomum cotylophorum*, *Moniezia benedeni*, *Setaria bidentata*, *Eucyathostomum longesubulatum*, *Strongyloides papillosus*, and *Nematodirus urichi*. *Setaria bidentate* was found in the omentum, and *Eucyathostomum longesubulatum* and an oxyurid-type worm was found in the large intestine [120,121,122]. In Brazil, parasites found in *Mazama americana* were *Dictyocaulus* spp., *Eucyathostomum* spp., *Eucyathostomum longibursatum*, *Seteria bidentate*, *Seteria* spp. *Haemonchus contortus*, *Haemonchus similis*, *Trichostrongylus axei*, *Trichostrongylus colubriformis*, *Physocephalus sexulatus*, *P. lassencei*, *Pygarginema verrucosa*, *Paramphistomum* spp., *Eimeria* spp., and *Cooperia punctata* [27,61,123,124] (Table 6). Lux Hoppe et al. [125] found *Trichostrongylus axei*, *Haemonchus contortus*, *H. similis*, *Physocephalus lassancei*, and *Pygarginema verrucosa* in the abomasum. *Capillaria bovis*, *Bunostomum phlebotomum*, and *Cooperia punctata* inhabited the small intestine.

In Mexico, *Paramphistomum cervi* was found in the rumen and abomasum as well as *Eimeria* spp., *Trichuris* spp., *Strongyloides* spp., and *Mammomonogamus* spp. [115,116] (Table 6). In Peru, the cysticercus of *Taenia hydatigena* was found in the omentum of *M. americana* as an intermediate host, while the definitive hosts were identified as carnivores [126]. A summary of endoparasites found in specific neo-tropical host species was provided (Table 7). Endoparasites that were common to different animal (host) was also listed (Table 7).

## 8. Conclusions

Most endoparasites were detected (clinical or subclinical) in the agouti, while for the red brocket deer (*Mazama americana*) few endoparasites were found (Table 7). This was in relation to the number of studies performed on these animals. A large majority of the authors viewed these neotropical non-domesticated animals as parasitic reservoirs that could affect domesticated introducted livestock species. The endoparasites of zoonotic significance found in the non-domesticatic neotropical animals were *Echinoccocus* spp., *Trichuris* spp., *Giardia* spp., and *Cryptosporidium* spp. There were a few reports of parasites showing negative effects on neotropical non-domesticated animals. Further studies on the effect of parasitism on these six neotropical species should elucidate their importance as pathogens, and their effect on the growth and performance of animals. Furthermore, the relation between the infestation grade and clinical manifestation should be deteremined.

## Figures and Tables

**Table 1:** Endoparasites found in the agouti (*Dasypocta* spp.) at different locations and points in time.

Parasites	Location	Effect on Animal or Pathological Lesion	Diagnostic	Year	Ref.
*Strongyloides* spp.	Trinidad	Not recorded	Necropsy and microscopy	1940	[20]
*Stichorchis giganteus*, *Raillietina demerariensis* var. *trinitatae*, *Strongyloides agouti*, *Trichuris gracilis* var. *trinitatae*, *Acanthocheilonema* spp., *Fuellebornema agoutii*, *Pudica pudica*, *Aspidodera binansata* var. *agoutiae*, *Helminthoxys urichi*	Trinidad	Not recorded	Necropsy and microscopy	1951	[21]
*Pudica pudica*, *Pudica gonosoma*, *Durettestrongylus baudi*, *Fuellebornema granulosa*, *Fuellebornema demarsae*	Paraguay	Not recorded	Necropsy and microscopy	1991	[25]
*Trichuris* spp., *Eucyathostomum copulatum*, *Helminthoxys urichi*, *Physocephalus meridionalis*, *Dipetalonema* spp.	Brazil	Not recorded	Necropsy and microscopy	2002	[26]
*Vianella trichospicula*, *Avellaria intermedia*	Brazil	Not recorded	Necropsy and microscopy	2006	[28]
Protozoan oocysts, *Trichuris* spp., *Strongyloides* spp.	Brazil	Not recorded	Coproscopy	2006	[29]
*Trichuris gracilis*, *Helminthoxys urichi*, *Physaloptera torresi*, *P. mediospiralis*, *Raillietina trinitatae*	Brazil	Not recorded	Necropsy, fecal floatation and microscopy	2006	[30]
*E. aguti*, *E. cotiae*, *E. paraensis*	Brazil	None of the infected animals showed signs of illness and faecal pellets were of normal consistency	Fecal floatation	2007	[31]
*Freitastrongylus angelae*	Brazil	Not recorded	Necropsy and microscopy	2007	[32]
*Strongyloides agouti*, *Paraspidodera uncinata*, *Helminthoxys urichi*, Pudicinae, *Trichuris gracilis*	Brazil	Not recorded	Necropsy and microscopy	2008	[33]
Strongylida, Rhabditidia, *Giardia* spp., *Eimeria* spp.	Brazil	Not recorded	Fecal floatation	2008	[34]
*Echinococcus oligarthrus* (hydatid cyst), strongyles, *Strongyloides* spp.	Guyana	Clinically healthy with no significant findings on bloodwork	Necropsy and microscopy	2009	[36]
*Trichuris gracilis* var. *trintatae*, *Strongyloides agouti*, *Helminthoxys urichi*, *Eimeria* spp., ascarid-like ova (unidentified species), trematode (unidentified species), cestode (unidentified species)	Trinidad	Presumed to the healthy animals	Necropsy, fecal floatation and microscopy	2016	[22]
*Eucyathostomum copulatum*	Brazil	Not recorded	Necropsy and microscopy	2016	[35]
*Strongyloides* spp., *Trichuris* spp., *Paraspidodera uncinata*	Trinidad	All animals were in good body condition (average score of 3 out of 5) and no history of gastrointestinal illness	Fecal floatation	2017	[23]
*Trichuris* spp. (2.2 × 10^2^ eggs/gram), *Strongyloides* spp. (4.28 × 10^2^ eggs/gram), *Eimeria* spp. (2.15 × 10^3^ oocysts/gram)	Trinidad	No signs of gastrointestinal illness and animals having an average body condition score of 3 out of 5	Fecal floatation (Mc Master)	2018	[24]

**Table 2:** Endoparasites found in the lappe (*Cuniculus paca/Agouti paca*) at different locations.

Parasites	Location	Effect on Animal or Pathological Lesion	Diagnosis	Year	References
*Heligmostrongylus sedecimradiatus*, *Longistriata breviscapula*, *Oswaldonema cruzi*, *Oswaldonema skrjabini*, *Paraspidodera uncinata*, *Paraspidodera* spp., *Pereiraia* spp., *Turgida torresi*, *Trichuris* spp., *Vianella avellari*	Brazil	Not recorded	Necropsy and microscopy	1997	[27]
*Strongyloides* spp., *Strongylus* spp., *Heligmostrongylus agouti*, *Physocephalus meridionalis*	Brazil	Not recorded	Necropsy and microscopy	2002	[26]
*Echinococcus vogeli*, *Calodium hepaticum*	Brazil	Liver fibrosis with an infiltrate of lymphocytes and macrophages	Necropsy and microscopy	2013	[39]
*Physocephalus* spp.	Brazil (Tocantins)	Congestion, interstitial hemorrhaging, and mononuclear infiltrates	Necropsy and microscopy	2012	[40]
*Eimeria* spp., *Strongyloides* spp., *Trichuris* spp., *Hymenolepis diminuta*	Brazil	Not recorded	Fecal floatation	2018	[41]
*Strongyloides* spp.	Brazil (Sao Luis)	Not recorded	Necropsy and microscopy	2018	[43]
*Strongyloides* spp., Strongyloidea, *Eimeria agouti*, *Capillaria* spp., Ascaroidea, *Taenia* spp., *Trichuris* spp.	Costa Rica	Not recorded	Necropsy and microscopy	1991	[44]
*Strongyloides* spp. Strongylida, *Trichuris* spp., Eucoccidia	Mexico	Not recorded	Coproscopy	2001	[45]
*Raillietina demerariensis*	Venezuela	Not recorded	Necropsy and microscopy	1988	[46]
*Echinococcus* spp.	Columbia	Cysts found in liver and peritoneum	Necropsy and microscopy	1979	[47]
*Echinococcus vogeli*	Bolivia	Not recorded	Necropsy and microscopy	1988, 2013	[48,49]

**Table 3:** Endoparasites found in the Capybara (*Hydrochoeris hydrochaeris*) at different locations.

Parasite	Location	Effect on Animal Health or Pathological Lesion	Diagnosis	Year	Ref.
*Taxorchis schistocotyle*	Panama	No lesions were observed in the intestinal tract	Necropsy and microscopy	1935	[57]
*Balantidium coli*	Florida	Ulceration on colon with infiltration of plasma cells, eosinophils and macrophages	Necropsy and histology	1961	[58]
*Taxorchis schostocotyle*, *Protozoophaga obesa*	Brazil	Not recorded	Necropsy and microscopy	2016	[35]
*Cruorifilaria tuberocauda*, *Eucoleus hydrochoeri*, *Echinocoleus hydrochaeri*, *Habronema clarki*, *Hydrochoerisnema anomalobursata*, *Protozoophaga obesa*, *Strongyloides chapini*, *Trichostrongylus axei*, *Vianella hydrochoeri*, *Yatesia hydrochoerus*	Brazil	Not recorded	Necropsy and microscopy	1997	[27]
*Trichostrongylus axei*, *Vianella hydrochoeri*, *Strongyloides chapini*, *Yatesia hydrochoerus*, *Cruorifilaria tubercauda*, *Capillaria hydrochoeri*, *Protozoophaga obesa*, *Taxorchis schistocotyle*, *Hippocrepis hippocrepis*, *Nudacotyle tertius*, *Monoecocestus hydrochoeri*, *Monoecocestus hagmanni*	Brazil	Not recorded	Necropsy and microscopy	1994	[69]
*Monoecocestus hagmanni*, *M. hydrochoeri*, *M. macrobursatum*, *Habronema clarki*, *Vianella hydochoeri*, *Protozoophaga obesa*, *Taxorchis schistocotyle*, *Hippocrepis hippocrepis*	Bolivia	Not recorded	Necropsy and microscopy	1995	[80]
*Eimeria trinidadensis*, *E. ichiloensis*, *E. boliviensis*	Bolivia and Venezuela	Not recorded	Necropsy and microscopy	1995	[81]
*Protozoophaga obesa*, *Viannella hydrochoeri*, *Hydrochoerisnema anomalobursata*, *Taxorchis schistocotyle*, *Hippocrepis hippocrepis*, *Capillaria hydrochoeri*, *Strongyloides chapini*, *Monoecocestus macrobursatum*, *M. hydrochoeri*, *M. hagmani*, *Trichostrongylus axei*, *Neocotyle neocotyle*, *Nudacotyle valdevaginatus*, *N. tertius*	Brazil	Not recorded	Necropsy and microscopy	2002	[62]
*Stongyloides* spp., *Habronema* spp., *Capillaria hysrochoeri*, *Vianella hydrochoeri*, *Protozoophaga obesa*, *Taxorchis schistocotyle*, *Hydrochoeristrema cabrali*, *Monoecocestus hydrochoeris, Monoecocestus hagmani*	Brazil	Not recorded	Necropsy and microscopy	2004	[70]
*Eimeria* spp., *Blastocystis* spp., *Balantidium coli*, Ascaridia, Trichostrongylidae, *Protozoophaga obesa*, *Strongyloides* spp., *Capillaria hydrochoaeri*, *Monoecocestus hydrochoeri*, *Taxorchis schistocotyle*	Argentina	Not recorded	Fecal floatation and sedimentation	2011	[76]
*Echinocoleus hydrochoeri*	Argentina	Not recorded	Necropsy and microscopy	2013	[78]
*Protozoophaga* spp., *Vianella* spp., *Strongyloides* spp., Ancylostomatidae	Brazil	Haematology and serum biochemistry showed a mild mal-nutrition and parasitism but no overt clinical signs were seen	Fecal floatation	2014	[75]
*Sarcocystis* spp., *Eimeria* spp., *Giardia* spp., *Cycloposthium hydrochaeri*, *C. incurvum*, *C. minutum*, *C. lenticularis*, *C. compressum*	Columbia	Not recorded	Fecal floatation, fecal sedimentation	2015	[83]
*Hippocrepis hippocrepis*, *H. fuelleborni*, *Philophthalmus lachrymosus*, *Taxorchis schistocotyle*, *Monoecocestus macrobursatus*, *M. hydrochaeri*, *Monoecocustus* spp., Anoplochephalidae, *Protozoophaga obesa*, *Strongyloides* spp.	Brazil	Not recorded	Necropsy and microscopy	2015	[66]
*Capillaria hydrochoeri*, *Hydrochoerisnema anomalobursata*, *Monoecocestus hagmanni*, *M. macrobursatum*, *Protozoophaga obesa*	Brazil	Not recorded	Necropsy and microscopy	2016	[71]
*Fasciola hepatica*, *Capillaria* spp.	Brazil	Not recorded	Coproscopy	2018	[68]
*Trichuris cutillasae*	Argentina	Not recorded	Molecular and Morphological techniques	2019	[79]

**Table 4:** Endoparasites found in the Manicou (*Didelphis marsupialis insularis*/*D. marsupialis*) at different locations.

Parasite	Location	Effect on Animal Health or Pathological Lesion	Diagnosis	Year	Ref.
*Aspidodera raillieti*, *Sublura trinitatis*, *Trichuris reesali*, *Trichuris minuta Trichuris urichi*, *Cruzia cameroni*, *Longistriata didelphis*, *Viannnaia hamata*, *Fuellebornema agouti*, *Delicata* spp., *Camerostrongylus didelphis*, *Capillaria* spp., *Physaloptera turgida*, *Spirocerca cylicola*, *Helminthoxys urichi*	Trinidad	Not recorded	Necropsy and microscopy	1951	[88]
*Didelphonema longispiculata* (syn. *Spirocerca cylicola*)	Trinidad	Not recorded	Necropsy and microscopy	1953	[87]
*Achillurabainia recondita*, *Rhopalias coronatus, Metadelphis evandroi*	Trinidad	Not recorded	Necropsy and microscopy	1958	[89]
*Isospora boughtoni*, *Isospora* spp.	Alabama	Not recorded	Sedimentation techniques	1969	[90]
*Phyaloptera turgida*, *Brachylaima virginianum*, *Cruzia americana*, *Capillaria aerophilia, Perostrongylus* spp.	Georgia	Weak, emaciated, circling and uncoordinated	Necropsy and microscopy	1975	[91]
*Didelphostrongylus hayesi*	Georgia	Not recorded	Necropsy and microscopy	1976	[92]
*Besnoitia* spp.	Brazil	Not recorded	Necropsy and Histology	1983	[93]
*Aspidodera raillieti*, *Cruzia tentaculata*, *Lagochilascaris turgida*, *Viannaia hamata*	Brazil	Not recorded	Microscopy	1997	[27]
*Capillaria* spp., *Eucoleus fluminensis*, *Trichuris minuta*, *Heterostrongylus* spp., *Mammomonogamus laryngeus*, *Aspidodera raillieti*, *Aspidodera* spp., *Cruzia tentaculata*, *Turgida turgida*, *Thelazia iheringi*, *Dipetalonema* spp., *Litomosoides* spp.	Brazil	Not recorded	Necropsy and Microscopy	2002	[26]
*Eimeria marmosopos*	Costa Rica	Not recorded	Fecal floatation and microscopy	2015	[96]
*E. marmosopos* (1 × 10^5^ oocyst/gram)	Costa Rica	Necrosis of cells of the small intestine	Histology	2015	[97]
*Amphimerus caudaletestis*, *Philandrophilus magnacirrus*, *Rhopalias coronatus*, *R. macracanthus*, *Thaumasioscolex didelphidis*, *Viannaia viannai*, *Travassostrongylus* spp., *Onicola luehei*, *Cruzia tentaculata*, *Trichuris didelphis*, *Turgida turgida*, *Capillaria* spp.	Mexico	Not recorded	Necropsy and Microscopy	2015	[98]
*Aspidodera* spp., *Cruzia tentaculata*	Brazil	Not recorded	Necropsy and microscopy	2016	[35]

**Table 5:** Endoparasites found in the collared peccary (*Tayassu tajacu*) at different locations.

Parasite	Location	Effect on Animal Health or Pathological Lesion	Diagnostics	Year	Ref.
*Moneizia bendeni*	Texas	Not recorded	Necropsy	1931	[99]
*Physocephalus sexalatus*, *Moniezia benedeni*, *Parostertagia heterospiculum*	Texas	Not recorded	Necropsy	1933	[100]
*Texicospirura turki*	Texas and New Mexico	Not recorded	Necropsy	1966	[110]
*Balantidium* spp., *Dirofilaria acutiuscula*, *Gongylonema baylisi*, *Parabronema pecariae*, *Parostertagia heterospiculum*, *Physocephalus* spp., *Texicospirura turki*, *Moniezia benedeni*, *Fascioloides magna*	Texas	Not recorded	Necropsy	1970	[101]
*Gongylonema pulchrum*, *Parabronema pecariae*, *Texicospirura turki*, *Physocephalus sexalatus* and *Moniezia benedeni*	Texas	No evidence of disease or loss of body condition	Necropsy	1985	[102]
*Eimeria chaparralensis*, *Eimeria dicotylensis*, *Eimeria pecari*, *Eimeria* spp., *Klossia* spp.	Texas	Not recorded	Fecal floatation	1996	[103]
*Ascarid suum*	Texas	Respiratory distress and coughing	Necropsy	1984	[117]
*Parabronema peccariae*, *Trichostrongylus columbiformis*	Mexico	Not recorded	Necropsy	1968	[105]
*Globocephalus urosubulatus*, *Parabronema pecariae*, *Parostertagia* spp., *Texicospiruria turki*, *Paramphistomum* spp., *Oesophagostomum* spp., *Moniezia benedeni*	Mexico	Not recorded	Fecal floatation, sedimentation and necropsy	2008	[113]
*Gonglyonema baylisi*	Brazil	Not recorded	Necropsy	1937	[107]
*Dirofilaria acutiuscula*, *Eucyathostomum dentatum*, *Gongylonema baylisi*, *Molineus semicircularis*, *Nematodirus molina*, *Oesophagostomum dentatum*	Brazil	Not recorded	Necropsy	1997	[27]
*Parabronema pecariae*	Brazil	Not recorded	Necropsy	2000	[109]
*Strongyloides* spp., *Eimeria* spp., *Isospora* spp., *Oesophagostomum* spp.	Brazil	Not recorded	Fecal centrifugation floatation and Mc Master Technique	2014	[114]
*Texicospiruria turki*, *Monodontus angulari*, *Eucyathostomum* spp., *Stichorchis giganteus*, *Moniezia benedeni*, *Ascaris* spp., *Eimeria* spp.	Bolivia	Not recorded	Fecal floatation and sedimentation	2014	[116]
*Entamoeba coli*, *Entamoeba* spp., *Cryptosporidium* spp., *Endolimax nana*, *Strongyloides* spp.	Panama	Not recorded	Fecal centrifugation floatation	2010	[115]
*Ascaris* spp., ancylostomatids, spirurid, *Paragonimus* spp.	Peru	Not recorded	Fecal floatational and sedimentation	2008	[110]
*Texicospiruria turki*, *Parabronema pecariae, Physocephalus sexalatus* and *Gongylonema* spp., *Monodontus semicircularis*, *Moniezia benedeni*, *Eucyathostomum dentatum*, *Trichuris* spp., *Balantidium coli*	Central Amazon	Not recorded	Necropsy	1986	[108]
*Toxocara alienate*	Surinam	Not recorded	Necropsy	1982	[112]
*Giardia* spp., *Cryptosporidium* spp., *Balantidium* spp., *Eimeria* spp.	Brazil	Not recorded	Fecal floatation	2010	[111]

**Table 6:** Endoparasites found in the Red Brocket deer (*Mazama americana*) at different locations.

Parasites	Location	Effect on Animal Health or Pathological Lesion	Diagnostic	Year	Ref.
*Mazamanema longibursatum*, *Ierestrongylus filiformis*, *Mazamastrongylus trinitatis*, *Nematodirus urichi*	Trinidad	Not recorded	Necropsy	1935	[120]
*Eucyathostomum longesubulatum, Setaria bidentata*, Oxyurid	Trinidad	Not recorded	Necropsy	1936	[121]
*Paramphistomum cotylophorum*, *Moniezia benedeni*, *Setaria bidentata*, *Eucyathostomum longesubulatum*, *Nematodirus urichi*, *Mazamastrongylus trinitatis*, *Strongyloides papillosus*	Trinidad	Not recorded	Necropsy	1936	[122]
*Dictyocaulus* spp., *Eucyathostomum* spp., *Eucyathostomum longibursatum*, *Seteria bidentata, Seteria* spp.	Brazil	Not recorded	Necropsy	1997	[27]
*Haemonchus contortus*, *Haemonchus similis*, *Trichostrongylus axei*, *Trichostrongylus colubriformis*, *Cooperia punctata*	Brazil	Not recorded	Necropsy	2000	[123]
*Paramphistomum* spp., *Eimeria* spp.	Brazil	Not recorded	Fecal floatation and sedimentation	2007	[124]
*Trichostrongylus axei*, *Haemonchus contortus*, *H. similis*, *Physocephalus lassancei, Pygarginema verrucosa, Capillaria bovis*, *Bunostomum phlebotomum*, *Cooperia punctata*	Brazil	Not recorded	Necropsy	2010	[125]
*Cooperia punctata*, *Eucyathostomum* spp., *Haemonchus contortus*, *H. similis*, *Physocephalus sexulatus*, *P. lassencei*, *Setaria bedentata*, *Trichostrongylus axei*, *T. colubriformis*, *Pygarginema verrucosa*	Brazil	Not recorded	Necropsy	2017	[61]
*Paramphistomum cervi*	Mexico	Not recorded	Necropsy	2008	[115]
*Eimeria* spp. and *Trichuris* spp., *Strongyloides* spp., *Mammomonogamus* spp.	Mexico	Not recorded	Fecal floatation and Necropsy	2014	[116]
*Taeinia hydatigena*	Peru	Not recorded	Necropsy	2015	[126]

**Table 7:** Endoparasites found in selected non-domesticated neotropical animals.

Neotropical Animals Species (Hosts)	Total No. of Internal Parasites Reported	Endoparasites Found in the Six Selected Host Species	Similar Endoparasites Found in Various Neotropical Animal Host Species
**Agouti (*D. leporina*)**	29	*Strongyloides* spp., *Stichorchis giganteus*, *Raillietina demerariensis* var. *trinitatae*, *Strongyloides agouti*, *Trichuris gracilis* var. *trinitatae*, *Trichuris* spp., *Acanthocheilonema* spp., *Fuellebornema agouti*, *Pudica pudica*, *Aspidodera binansata* var. *agouti*, *Helminthoxys urichi*, *Paraspidodera uncinata*, *Pudica gonosoma*, *Durettestrongylus baudi*, *Fuellebornema granulosa*, *Fuellebornema demarsae*, *Eucyathostomum copulatum*, *Physocephalus meridonalis*, *Physocephalus torresi*, *Physocephalus mediospiralis*, *Diptalonema* spp., *Vianella trichospicula*, *Avellaria intermedia*, *Eimeria aguti*, *Eimeria cotiae*, *Eimeria paraensis, Eimeria spp.*, *Giardia* spp., *Echinococcus oligarthrus*, *Freitastrongylus angelae*	*Helminthoxys urichi* (manicou and agouti) *Paraspidodera uncinata, Trichuris* spp., *Strongyloides* spp., *Physocephalus meridonalis, Eimeria agouti, Raillietina demarariensis, Eimeria spp.*, (agouti and lappe)
**Lappe (*A. paca*)**	24	*Heligmostrongylus sedecimradiatus*, *Heligmostrongylus agouti*, *Longistriata breviscapula*, *Oswaldonema cruzi*, *Oswaldonema skrjabini*, *Paraspidodera uncinata*, *Paraspidodera* spp., *Pereiraia* spp., *Turgida torresi*, *Trichuris* spp., *Vianella avellari*, *Strongyloides* spp., *Strongylus* spp., *Physocephalus meridonalis*, *Echinococcus vogeli*, *Coladium hepaticum*, *Physocephalus* spp., *Eimeria* spp., *Hymenolepis diminuta*, *Eimeria agouti*, *Capillaria* spp., *Taenia* spp., *Raillietina demarariensis*, *Echinococcus* spp.	*Strongyloides* spp., *Giardia* spp., *Eimeria* spp. (agouti, lappe, capybara, and collared peccary)
**Capybara (*H. hydrochaeris*)**	42	*Taxorchis schistocotyle*, *Balantidium coli*, *Protozoophaga obesa*, *Cruorifilaria tuberocauda*, *Eucoleus hydrochoeri*, *Echinocoleus hydrochaeri*, *Habronema clarki*, *Habronema* spp., *Hydrochoerisnema anomalobursata*, *Strongyloides chapini*, *Strongyloides* spp., *Trichostrongylus axei*, *Vianella hydrochoeri*, *Vianella* spp., *Yatesia hydrochoerus*, *Hippocrepis hippocrepis*, *Hippocrepis fuelleborni*, *Philophthalmus lachrymosus*, *Neocotyle neocotyle*, *Nudacotyle valdevaginatus*, *Nudacotyle tertius*, *Monoecocestus hydrochoeri*, *Monoecocestus hagmanni*, *Monoecocestus macrobursatum*, *Monoecocestus* spp., *Eimeria trinidadensis*, *Eimeria ichiloensis*, *Eimeria boliviensis*, *Eimeria* spp., *Blastocystis* spp., *Sarcocystis* spp., *Giardia* spp., *Cycloposthium hydrochaeri*, *Cycloposthium incurvum*, *Cycloposthium minutum*, *Cyclposthium lenticularis*, *Cycloposthium compressum*, *Fasciola hepatica*, *Capillaria* spp., *Trichuris cutillasae*	*Trichostongylus columbiformis*, *Strongyloides* spp., *Eimeria* spp., *Physocephalus sexulatus*, *Trichuris* spp., *Moniezia benedeni*, *Paramphisotmum* spp. (capybara and red brocket deer) *Capillaria* spp. (capybara and manicou) *Balantidium coli*, *Giardia* spp., (common to capybara and collared peccary)
**Manicou (*D. marsupialis insularis*)**	44	*Aspidodera raillieti*, *Aspidodera* spp., *Sublura trinitatis*, *Trichuris reesali*, *Trichuris minuta*, *Trichuris urichi*, *Trichuris didelphis*, *Cruzia cameroni*, *Cruzia americana*, *Cruzia tentaculata*, *Longistrata didelphis*, *Viannaia hamata*, *Viannaia viannai, Fuellebornema agouti*, *Delicata* spp., *Travassostrongylus* spp., *Camerostrongylus didelphis*, *Capillaria aerophilia*, *Capillaria* spp., *Physaleptera turgida*, *Spirocerca cylicola*, *Helminthoxys urichi*, *Achillurabainia recondite*, *Rhopalias coronatus*, *Metadelphis evandroi*, *Isospora boughtoni*, *Isospora* spp., *Brachylaima virginianum*, *Perostrongylus* spp., *Heterostrongylus* spp., *Didelphostrongylus hayesi*, *Besnoitia* spp., *Lagochilascaris turgida*, *Eucoleus fluminensis*, *Mammomonogamus laryngeus*, *Turgida turgida*, *Thelazia iheringi*, *Diptalonema* spp., *Litomosoides* spp., *Eimeria marmosopos*, *Amphimerus caudaletestis*, *Philandrophilus magnacirrus*, *Thaumasioscolex didelphidis*, *Onicola luehei*	*Trichostongylus columbiformis*, *Strongyloides* spp., *Eimeria* spp., *Physocephalus sexulatus*, *Trichuris* spp., *Moniezia benedeni*, *Paramphisotmum* spp. (capybara and red brocket deer) *Capillaria* spp. (capybara and manicou) *Balantidium coli*, *Giardia* spp., (common to capybara and collared peccary)
**Collared peccary (*T. tajacu*)**	39	*Moniezia benedeni*, *Physocephalus sexalatus*, *Physocephalus* spp., *Parostertagia heterospiculum*, *Texicospirura turki*, *Balantidium coli*, *Dirofilaria acutiuscula*, *Gongylonema baylisi*, *Gongylonema pulchrum*, *Parabronema pecariae*, *Fascioloides magna*, *Eimeria chaparrelensis*, *Eimeria dicotylensis, Eimeria pecari*, *Eimeria* spp., *Isospora* spp., *Klossia* spp., *Ascaris suum*, *Ascaris* spp., *Trichostrongylus columbiformis*, *Globocephalus urosubulatus*, *Paramphistomum* spp., *Oesophagostomum* spp., *Oesophagostomum dentatum*, *Nematodirus molina*, *Eucyathostomum dentatum*, *Eucyathostomum* spp., *Stichorchis giganteus*, *Molineus semicircularis*, *Strongyloides* spp., *Paragonimus* spp., *Monodontus angularis*, *Entamoeba coli*, *Entamoeba* spp., *Cryptosporidium* spp., *Endolimax nana*, *Trichuris* spp., *Toxocara alienata*, *Giardia* spp.	*Trichurs* spp., *Strongyloides* spp., *Eimeria* spp., *Giardia* spp., *Stichorchis giganteus* (agouti and collared peccary)
**Red Brocket deer (*M. americana*)**	30	*Mazamanema longibursatum*, *Ierestrongylus filiformis*, *Mazamastrongylus trinitatis*, *Nematodirus urichi*, *Eucyathostomum longesubulatum*, *Eucyathostomum* spp., *Setaria bidentata*, *Setaria* spp., *Paramphistomum cotylophorum*, *Paramphistomum cervi*, *Paramphistomum* spp., *Moniezia benedeni*, *Strongyloides papillosus*, *Strongyloides* spp., *Dictyocaulus* spp., *Haemonchus contortus*, *Haemonchus similis*, *Trichostrongylus axei*, *Trichostrongylus columbiformis*, *Cooperia punctata*, *Eimeria* spp., *Physocephalus lassancei*, *Physocephalus sexalatus, Pygarginema verrucosa*, *Capillaria bovis*, *Bunostomum phlebotomum*, *Trichuris* spp., *Taenia* spp., *Mammomonogamus* spp.	*Trichuris* spp., *Strongyloides* spp., *Eimeria* spp. (agouti and red brocket deer) *Strongyloides* spp., *Eimeria* spp. (lappe and red brocket deer)
